# Di-*tert*-butyl­diiso­thio­cyanato­tin(IV)

**DOI:** 10.1107/S2414314624012446

**Published:** 2025-01-07

**Authors:** Anne Kamrowski, Hans Reuter

**Affiliations:** a Chemistry, Osnabrück University, Barabarstr. 7, 49069 Osnabrück, Germany; University of Aberdeen, United Kingdom

**Keywords:** crystal structure, iso­thio­cyanate, inter­molecular inter­actions, spherical coordinates, titling

## Abstract

The title compound, [Sn(C_4_H_9_)_2_(NCS)_2_], which crystallizes with one and a half mol­ecules in the asymmetric unit, represents a new structure type for inter­molecular sulfur⋯tin inter­actions, which is characterized by an anti­parallel (*A*) arrangement of the dipole moments of the individual mol­ecules. In the resulting zigzag chains, the mol­ecules are related to each other by mirror planes (*m*) and twofold rotation axes (2), both perpendicular to the propagation plane, while translation is realized *via* a glide plane in direction of the crystallographic *c* axis, a combination of symmetry elements unique in the structural chemistry of diorganotin(IV) dihalides and pseudohalides, *R*_2_Sn*X*_2_ with *X* = Hal or NCS. Its characteristics are subsumed in the term *Am*2*c* for this kind of inter­molecular association pattern. The tilting of the NSnN-planes in relation to the propagation plane is described in terms of spherical coordinates.

## Abstract 2

Di-*tert*-butyl­diiso­thio­cyanato­tin(IV), ^*t*^Bu_2_Sn(NCS)_2_, exhibits a new kind of inter­molecular association *via* Sn⋯S inter­actions resulting in an anti­parallel, zigzag arrangement of the mol­ecular dipole moments. The chain-like structure motif is classified as *Am*2*c* according to its structure determining symmetry elements.

## Graphical abstract

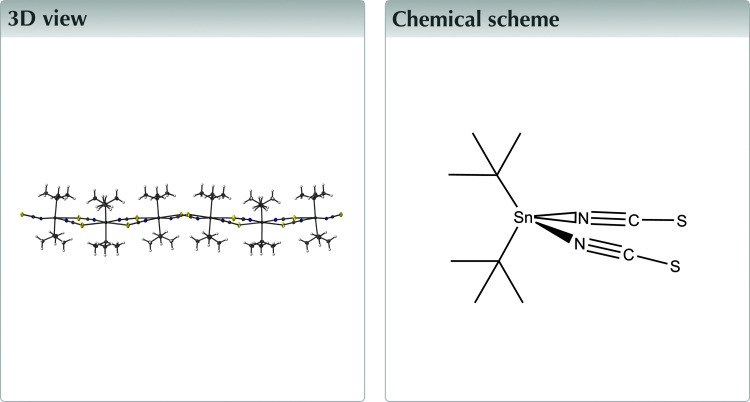

## Structure description

As linear, polyatomic pseudo-halide ion, the thio­cyanate ion, NCS^−^, is able to replace mono-atomic, spherical halide atoms in many compounds, which in the case of diorgano­tin(IV) dihalides, *R*_2_SnHal_2_, leads to the formation of the so-called diorganotin(IV) diiso­thio­cynates, *R*_2_Sn(NCS)_2_, as the pseudo-halide ion binds to the ‘*hard*’ tin atom *via* its ‘*hard*’ nitro­gen atom in accordance with the HSAB principle.

In terms of structural chemistry these compounds are of special inter­est with regard to their inter­molecular inter­actions, which for steric reasons can only take place *via* the ‘*soft*’ sulfur atoms. In case of the methyl and ethyl compounds (Britton, 2006 ▸), these inter­actions result in a chain-like arrangement of the individual mol­ecules with a parallel orientation of their dipole moments while the phenyl compound (Pancratz *et al.*, 2024 ▸) represents a di-periodic coordination polymer in which the mol­ecules have lost their individuality. In search of a mol­ecular *diiso­cyanate* we have prepared for the first time the title *tert*-butyl compound because the bulky *tert*-butyl substituents prevent an inter­molecular association in the comparable *dichloride* (Dakternieks *et al.*, 1994 ▸).

**Figure 1:**
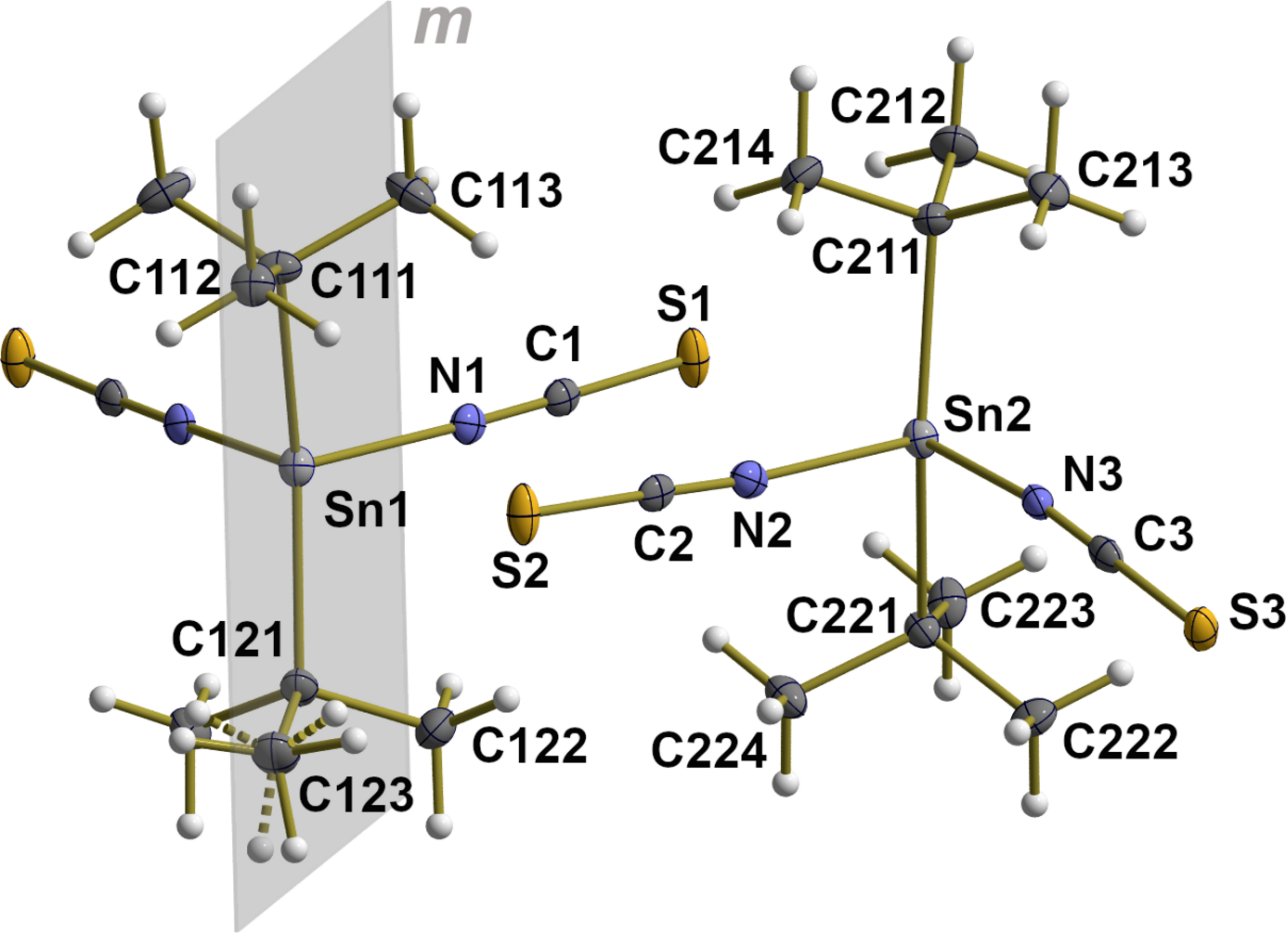
Ball-and-stick model of the tetra­hedral environment of the two crystallographically independent ^*t*^Bu_2_Sn(NCS)_2_ mol­ecules with atom numbering given for the asymmetric unit and orientation of the crystallographic mirror plane, *m*, in the mol­ecule of Sn1. With the exception of the hydrogen atoms, which are shown as spheres of arbitrary radius, all other atoms are drawn as displacement ellipsoids at the 40% probability level. Disorder of the hydrogen atoms attached to C123 is shown by dashed bonds in the case of the second hydrogen-atom orientation.

The title compound, ^*t*^Bu_2_Sn(NCS)_2_, crystallizes in the ortho­rhom­bic space group *Pbcm* with 12 mol­ecules in the unit cell and one and a half mol­ecules in the asymmetric unit (Fig. 1 ▸). The half mol­ecule results from a crystallographic mirror plane that bis­ects the tin atom and the two *tert*-butyl groups with order/disorder of the hydrogen atoms of the affected methyl group.

The carbon–carbon bond lengths within the *tert*-butyl groups [C—C = 1.514 (7)–1.530 (4) Å, mean value = 1.525 (6) Å] are only slightly shorter than the value given in literature [*d*(C*sp*^3^—CH_3_) = 1.534 (11) Å (Allen *et al.*, 1987 ▸)]. The mean bond angles of 110.1 (4)° between the methyl groups correspond very well with tetra­hedrally coordinated, *sp*^3^-hybridized carbon atoms. With the tin–carbon–carbon bond angles, it is noticeable that in each *tert*-butyl group two angles are smaller [mean value: 107.1 (4)°] than the third one [mean value: 110.1 (9)°].

The bond lengths and angles describing the coordination sphere of the tin atoms, however, have very unusual values. Thus, the Sn—C distances of 2.214 (4)–2.227 (3) Å [mean value: 2.221 (6) Å] are quite long and the bond angles of 157.3 (2)/157.2 (2)° between the *tert*-butyl groups are greatly widened in comparison with the corresponding values [2.149 (4)/2.151 (4) Å, 133.1 (2)°] in the crystal structure of the parent compound di-*tert*-butyl­tin(IV) dichloride, ^*t*^Bu_2_SnCl_2_ (Dakternieks *et al.*, 1994 ▸). The same applies to the bond angles between the inorganic ligands which are considerably smaller in the title compound [84.4 (1)°/84.7 (1)°] than in the dichloride [101.86 (5)°], an effect that can be attributed to the smaller size of the nitro­gen atoms in comparison with the chloride ions. Similar changes of bond angles are found in compounds *R*_2_Sn*X*_2_ with *R* = Me and Et, respectively when comparing *X* = Cl and *X* = NCS. For *R* = Me, 〈(C—Sn—C) changes from 142.2 (4)° for *X* = Cl (Reuter & Pawlak, 2001 ▸) to 147.6 (1)° (Britton, 2006 ▸) and 〈(*X*—Sn—*X*) from 98.60 (9)° to 86.08 (8)° for *X* = NCS (Britton, 2006 ▸ ▸)], and for *R* = Et, 〈(C—Sn—C) changes from 134.0 (6)° for *X* = Cl (Alcock & Sawyer, 1977 ▸) to 153.03 (6)° for *X* = NCS (Britton, 2006) and 〈(*X*—Sn—*X*) from 96.0 (1)° to 83.57 (8)° for *X* = NCS. The tin–carbon bond lengths, however, differ only slightly in the compounds in question.

The bond lengths [mean N—C = 1.103 (3) Å, mean C—S = 1.627 (4) Å] and angles [mean 〈(N—C—S = 178.5 (3)°] within the almost linear iso­thio­cyanate groups are only slightly affected by their coordination behavior (Table 1 ▸) and almost identical with the values found in the other structurally determined *diiso­thio­cyanates* [*R* = Me, Et (Britton, 2006 ▸), *R* = Ph (Pancratz *et al.*, 2024 ▸)]. These values correspond very well with a formal carbon–nitro­gen triple [*d*(C*sp*—N) = 1.155 (12) Å (Allen *et al.*, 1987 ▸)] and a carbon–sulfur single [*d*(C_*sp*_—S) = 1.630 (14) Å (Allen *et al.*, 1987 ▸)] bond. Their coordination to the tin atoms *via* the nitro­gen atoms is characterized by a mean tin–nitro­gen distance of 2.176 (5) Å over all four NCS groups but the Sn—N—C bond angles show a greater variance. Three of the four bond angles are around 174.5 (8)° while one only reaches 167.0 (2)° (Table 1 ▸).

**Figure 2:**
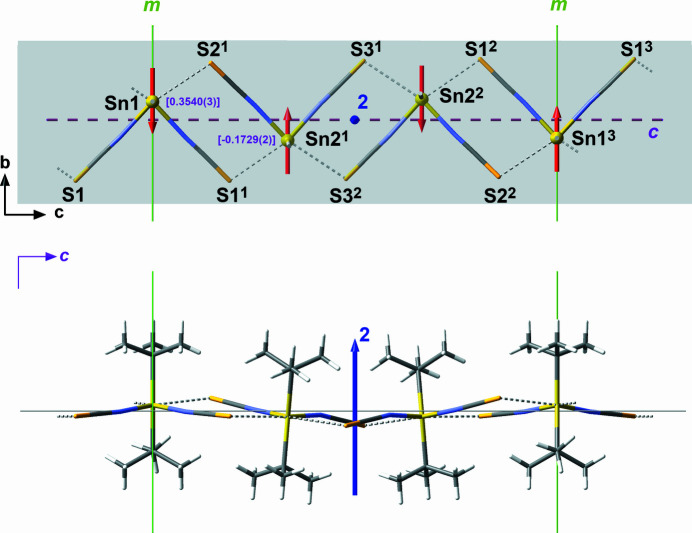
Schematic, ball-and-stick representation of the anti­parallel, chain-like arrangement of the dipole moments (red arrows of arbitrary units, orientation assumed in the direction of the center point between the two nitro­gen atoms) of the ^*t*^Bu_2_Sn(NCS)_2_ mol­ecules as a result of the tin⋯sulfur inter­actions (dashed sticks in gray) and their relation to the crystallographic symmetry elements: mirror plane = *m*, green line; twofold rotation axis perpendicular to the gray propagation plane = 2, blue arrow; axial glide plane = *c*, dashed line, violet; above = top view on the propagation plane with organic groups omitted for clarity, below = side view; values in square brackets = distances (Å) of the tin atoms from the glide plane; atom color code used: Sn = bronze, N = blue, C = black, S = yellow, H = white; symmetry transformations used to generate equivalent atoms: (1) *x*, *y*, 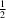 − *z*; (2) *x*, 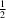 − *y*, 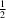 + *z*; (3) *x*, 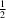 − *y*, 1 − *z*.

Despite the widening of the bond angles between the *tert*-butyl groups, inter­molecular tin–sulfur distances [3.1312 (9)–3.1519 (9) Å] are of the same order of magnitude as in the corresponding methyl [3.146 (1) Å] and ethyl [3.060 (1) Å] compounds (Britton, 2006 ▸) and thus significantly longer than in Ph_2_Sn(NCS_)2_ [2.7224 (5) Å; Pancratz *et al.*, 2024 ▸]. In relation to the sum (2.43 Å) of the covalent radii (Cordero *et al.*, 2008 ▸) of tin (1.39 Å) and sulfur (1.04 Å), these secondary tin–sulfur contact lengths of around 3.1 Å are quite long (+0.67 Å = + 28%) but in relation to the sum (3.97 Å) of the van der Waals radii (Mantina *et al.*, 2009 ▸) of tin (2.17 Å) and sulfur (1.80 Å) quite short (–0.87 Å = 22%). In summary, these secondary contacts lead to a chain-like arrangement of the individual mol­ecules with anti-parallel arrangement of the dipole moments and carbon–sulfur⋯tin angles of 99.8 (1)–100.7 (1)° (Fig. 2 ▸).

Within the zigzag-chains the two mol­ecules are related to each other by a sequence of mirror planes, *m*, and twofold rotation axes, 2, all perpendicular to the propagation plane parallel to the crystallographic *a* and *b* axes, while translation of the mol­ecules is arranged *via* the glide plane *c* from which the tin atoms are at different distances (Fig. 2 ▸). The repeat unit therefore corresponds to the length of the *c* axis = 36.0164 (9) Å. According to the classification scheme developed earlier (Ye & Reuter, 2012 ▸) based on the parallel (*P*) or anti-parallel (*A*) arrangement of the dipole moments and the symmetry elements involved in the inter­molecular association, the present structure type can be denoted as *Am*2*c*.

In the structural chemistry of diorganotin dihalides, *R*_2_SnHal_2_, anti-parallel arrangements of the dipole moments into zigzag chains are relatively common, but do not occur in the present combination (2, *m*) of symmetry elements: Me_2_SnCl_2_ = *Amm*2_1_ (Reuter & Pawlak, 2001 ▸), Et_2_SnBr_2_ = *A*22_1_ (Alcock & Saywer, 1977), ^*n*^Bu_2_SnCl_2_ = *A*2_1_ (Sawyer, 1988 ▸), Et_2_SnCl_2_ = *Ac* (Alcock & Sawyer, 1977 ▸).

**Figure 3:**
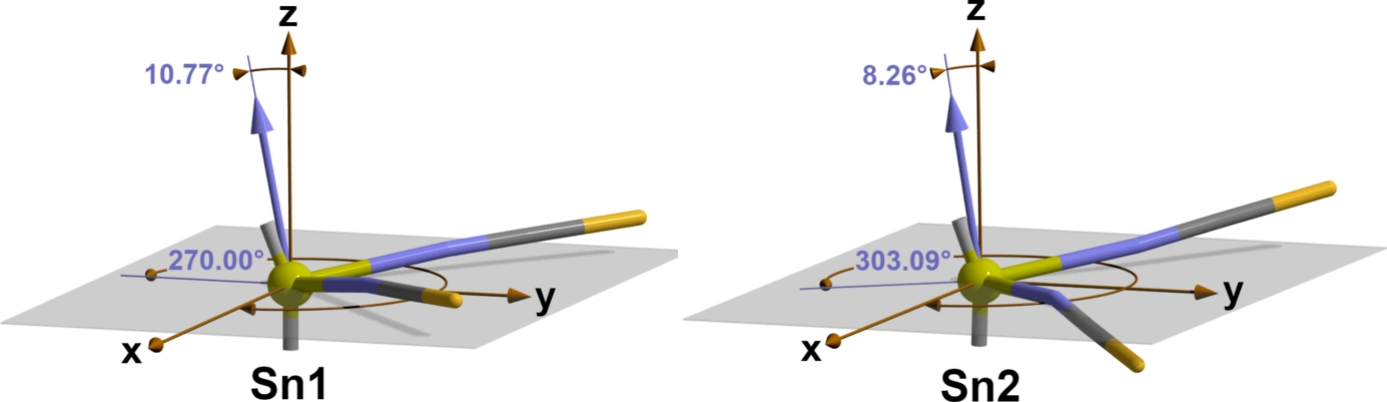
Spherical coordinate system used to calculate the spherical coordinates *Θ* and *φ* in order to characterize the tilting of the NSnN-planes of both mol­ecules in relation to the propagation plane (gray), arbitrary values for the authoritative normal vector *N*_NSnN_ (blue); for clarity the positions of the organic ligands are only indicated by short sticks.

As can easily be seen in Figs. 1 ▸ and 2 ▸, the N—Sn—N planes of the two mol­ecules are not coplanar to the propagation plane. Qu­anti­tatively, the out-of-plane orientation of these planes can be described in terms of spherical coordination systems defined by the radial distance *r*, the polar angle *Θ* and the azimuthal angle *φ* (Fig. 3 ▸): with the tin atom as origin, *r* as the lengths of the normal vector *N*_NSnN_, *Θ* as the angle between this normal vector and the polar axis *z* (= the crystallographic *a* axis) and *φ* as the angle of rotation around the polar axis *z* in the meridional *xy* plane (= plane through Sn and coplanar to the propagation plane *bc*). The corresponding values are *Θ* = 10.77°*, φ* = 270° for the NSnN plane of Sn1, and *Θ* = 8.26°, *φ* = 303.09° for the NSnN plane of Sn2.

**Figure 4:**
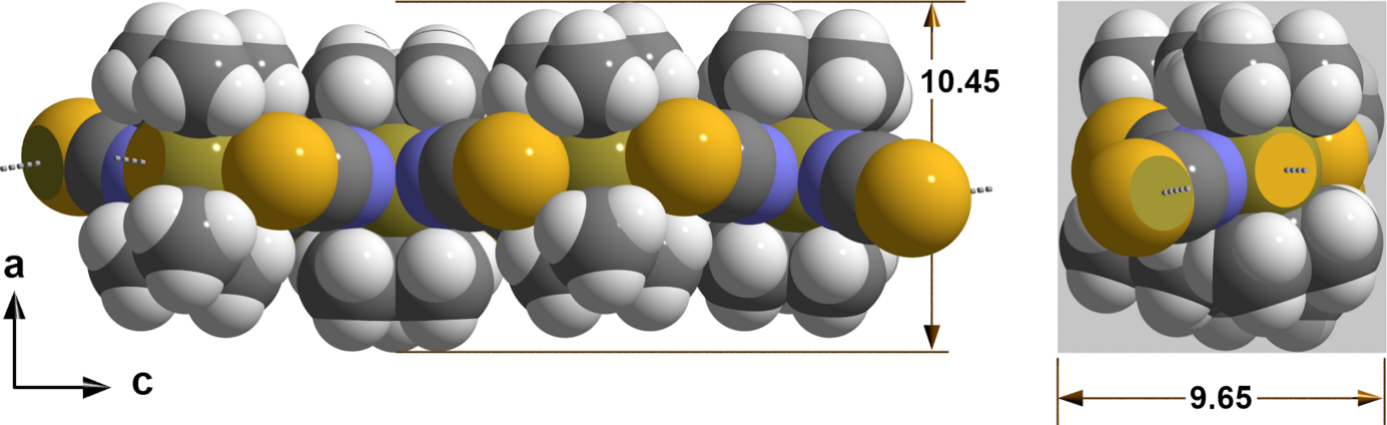
Detail of the chain-like arrangement of the ^*t*^Bu_2_Sn(NCS)_2_ mol­ecules as space-filling model with dimensions in Å; left side = side view looking down the *b* axis, right side = front view; atoms are represented as single-colored or truncated, two-colored spheres according to their van der Waals radii and cut-offs based on the inter­section of the two spheres with cut-off faces showing the color of the inter­penetrating atom, inter­molecular tin⋯sulfur contacts are visualized as dashed sticks in gray; atom color code used: Sn = bronze, N = blue, C = black, S = yellow, H = white.

**Figure 5:**
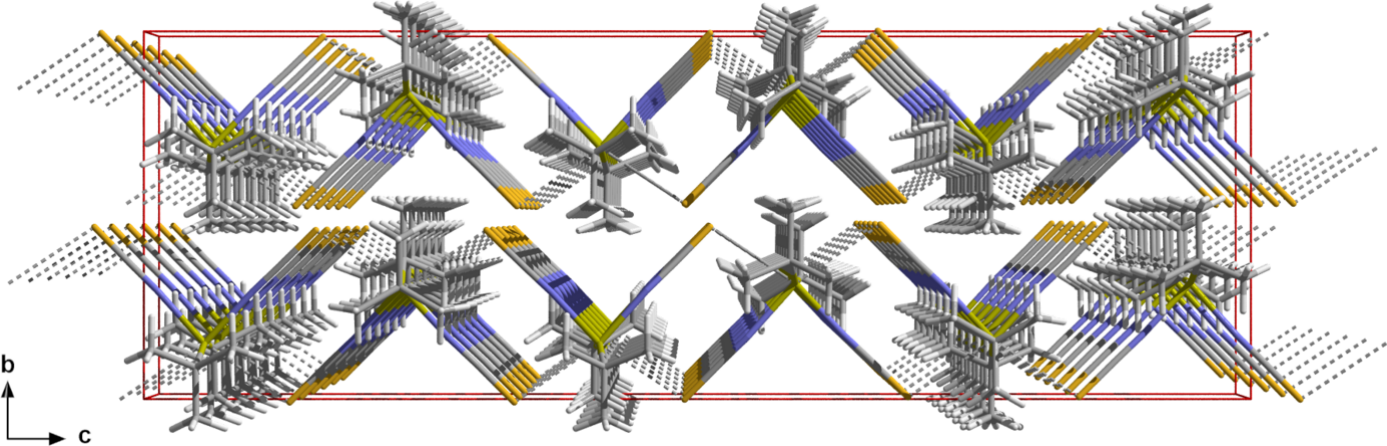
Ball-and-stick representation showing the mol­ecule packing and inter­locking of the chains formed when looking down the *a* axis; inter­molecular tin⋯sulfur contacts are shown as dashed sticks in gray.

Including the organic residuals into account the sinusoidal chains have an almost rectangular cross-section (Fig. 4 ▸) of about 9.65 × 10.45 Å and are arranged in the direction of the *b* axis whereby the wave crest of the one chain engaged in the wave valley of the other one (Fig. 5 ▸). For an inter­pretation of the secondary contacts in terms of 3*c*–4*e* bonds see Alcock & Sawyer (1977 ▸).

## Synthesis and crystallization

The synthesis was carried out according to a published protocol from sodium thio­cyanate and di-*tert*-butyl­tin(IV) dichloride, ^*t*^Bu_2_SnCl_2_, (Kandil & Allred, 1970 ▸) in ethanol (molar ratio 1:2): colorless, needle-like single crystals were obtained after recrystallization from toluene solution.

## Refinement

Crystal data, data collection and structure refinement details are summarized in Table 2 ▸.

## Supplementary Material

Crystal structure: contains datablock(s) I. DOI: 10.1107/S2414314624012446/hb4502sup1.cif

Structure factors: contains datablock(s) I. DOI: 10.1107/S2414314624012446/hb4502Isup2.hkl

CCDC reference: 2412795

Additional supporting information:  crystallographic information; 3D view; checkCIF report

## Figures and Tables

**Table 1:** Selected geometric parameters (Å, °)

Sn1—N1	2.180 (3)	Sn1—C111	2.224 (4)
Sn2—N2	2.172 (3)	S1—Sn2	3.1519 (9)
Sn2—N3	2.175 (3)	S2—Sn1	3.1312 (9)
Sn1—C121	2.214 (4)	S3—Sn2^i^	3.1355 (9)
			
C1—N1—Sn1	174.6 (2)	C3—N3—Sn2	167.0 (2)
C2—N2—Sn2	175.2 (3)		

Symmetry code: (i) 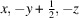.

**Table 2:** Experimental details

Crystal data	
Chemical formula	[Sn(C_4_H_9_)_2_(NCS)_2_]
*M*_r_	349.07
Crystal system, space group	Orthorhombic, *Pbcm*
Temperature (K)	100
*a*, *b*, *c* (Å)	9.6669 (3), 11.9440 (4), 36.0164 (9)
*V* (Å^3^)	4158.5 (2)
*Z*	12
Radiation type	Mo *K*α
μ (mm^−1^)	2.12
Crystal size (mm)	0.18 × 0.12 × 0.06

Data collection	
Diffractometer	Bruker APEXII CCD
Absorption correction	Multi-scan (*SADABS*; Krause *et al.*, 2015 ▸)
*T*_min_, *T*_max_	0.697, 0.891
No. of measured, independent and observed [*I* > 2σ(*I*)] reflections	169441, 5093, 4052
*R*_int_	0.104
(sin θ/λ)_max_ (Å^−1^)	0.661

Refinement	
*R*[*F*^2^ > 2σ(*F*^2^)], *wR*(*F*^2^), *S*	0.033, 0.067, 1.12
No. of reflections	5093
No. of parameters	225
H-atom treatment	H-atom parameters constrained
Δρ_max_, Δρ_min_ (e Å^−3^)	0.62, −1.01

Computer programs: *APEX2* and *SAINT* (Bruker, 2009 ▸), *SHELXS97* (Sheldrick 2008 ▸), *SHELXL2014/7* (Sheldrick, 2015 ▸), *DIAMOND* (Brandenburg, 2006 ▸), *Mercury* (Macrae *et al.* (2020 ▸), *POVRAY* (Povray, 2004 ▸) and *publCIF* (Westrip, 2010 ▸).

## full crystallographic data

### Di-tert-butyldiisothiocyanatotin(IV) Crystal data

**Table:**

[Sn(C_4_H_9_)_2_(NCS)_2_]	*D*_x_ = 1.673 Mg m^−^^3^
*M**_r_* = 349.07	Mo *K*α radiation, λ = 0.71073 Å
Orthorhombic, *P**b**c**m*	Cell parameters from 9996 reflections
*a* = 9.6669 (3) Å	θ = 2.4–29.2°
*b* = 11.9440 (4) Å	µ = 2.12 mm^−^^1^
*c* = 36.0164 (9) Å	*T* = 100 K
*V* = 4158.5 (2) Å^3^	Plate, colourless
*Z* = 12	0.18 × 0.12 × 0.06 mm
*F*(000) = 2088	

### Di-tert-butyldiisothiocyanatotin(IV) Data collection

**Table:**

Bruker APEXII CCD diffractometer	4052 reflections with *I* > 2σ(*I*)
φ and ω scans	*R*_int_ = 0.104
Absorption correction: multi-scan (SADABS; Krause *et al.*, 2015)	θ_max_ = 28.0°, θ_min_ = 2.1°
*T*_min_ = 0.697, *T*_max_ = 0.891	*h* = −12→12
169441 measured reflections	*k* = −15→15
5093 independent reflections	*l* = −47→47

### Di-tert-butyldiisothiocyanatotin(IV) Refinement

**Table:**

Refinement on *F*^2^	Hydrogen site location: mixed
Least-squares matrix: full	H-atom parameters constrained
*R*[*F*^2^ > 2σ(*F*^2^)] = 0.033	*w* = 1/[σ^2^(*F*_o_^2^) + (0.0162*P*)^2^ + 7.7574*P*] where *P* = (*F*_o_^2^ + 2*F*_c_^2^)/3
*wR*(*F*^2^) = 0.067	(Δ/σ)_max_ = 0.001
*S* = 1.12	Δρ_max_ = 0.62 e Å^−^^3^
5093 reflections	Δρ_min_ = −1.01 e Å^−^^3^
225 parameters	Extinction correction: *SHELXL2014/7* (Sheldrick 2015), Fc^*^=kFc[1+0.001xFc^2^λ^3^/sin(2θ)]^-1/4^
0 restraints	Extinction coefficient: 0.00019 (2)
Primary atom site location: structure-invariant direct methods	

### Di-tert-butyldiisothiocyanatotin(IV) Special details

**Table:**

Geometry. All esds (except the esd in the dihedral angle between two l.s. planes) are estimated using the full covariance matrix. The cell esds are taken into account individually in the estimation of esds in distances, angles and torsion angles; correlations between esds in cell parameters are only used when they are defined by crystal symmetry. An approximate (isotropic) treatment of cell esds is used for estimating esds involving l.s. planes.
Refinement. The H atoms were geometrically placed (C—H = 0.98 Å) and refined as riding atoms. The *U*_iso_ vaules for the H atoms were contrained to be the same for all atoms attached to a particular carbon atom.

### Di-tert-butyldiisothiocyanatotin(IV) Fractional atomic coordinates and isotropic or equivalent isotropic displacement parameters (Å^2^)

**Table:**

	*x*	*y*	*z*	*U*_iso_*/*U*_eq_	Occ. (<1)
Sn1	0.71338 (3)	0.31769 (2)	0.2500	0.01406 (8)	
C111	0.4833 (4)	0.3193 (4)	0.2500	0.0186 (9)	
C112	0.4302 (5)	0.4399 (4)	0.2500	0.0220 (10)	
H111	0.3289	0.4393	0.2500	0.030 (5)*	
H112	0.4636	0.4782	0.2277	0.030 (5)*	
C113	0.4340 (3)	0.2588 (3)	0.21517 (10)	0.0264 (8)	
H114	0.4663	0.1810	0.2157	0.030 (5)*	
H115	0.4713	0.2964	0.1932	0.030 (5)*	
H116	0.3327	0.2600	0.2142	0.030 (5)*	
C121	0.9254 (5)	0.3877 (4)	0.2500	0.0204 (10)	
C122	0.9991 (3)	0.3436 (3)	0.21538 (10)	0.0272 (8)	
H124	1.0943	0.3718	0.2149	0.039 (6)*	
H125	0.9500	0.3692	0.1931	0.039 (6)*	
H126	1.0002	0.2616	0.2159	0.039 (6)*	
C123	0.9251 (5)	0.5145 (4)	0.2500	0.0302 (11)	
H121	0.8625	0.5417	0.2694	0.039 (6)*	0.5
H122	0.8936	0.5418	0.2258	0.039 (6)*	0.5
H123	1.0189	0.5420	0.2548	0.039 (6)*	0.5
N1	0.7446 (3)	0.1848 (2)	0.20936 (8)	0.0187 (6)	
C1	0.7531 (3)	0.1200 (3)	0.18662 (9)	0.0173 (6)	
S1	0.76537 (10)	0.02369 (7)	0.15494 (2)	0.0272 (2)	
Sn2	0.76789 (2)	0.17360 (2)	0.08292 (2)	0.01418 (7)	
C211	0.5510 (3)	0.1151 (3)	0.07802 (9)	0.0199 (7)	
C212	0.5441 (4)	−0.0125 (3)	0.07864 (10)	0.0311 (8)	
H211	0.5816	−0.0401	0.1022	0.037 (4)*	
H212	0.5986	−0.0427	0.0580	0.037 (4)*	
H213	0.4476	−0.0365	0.0761	0.037 (4)*	
C213	0.4912 (3)	0.1599 (3)	0.04175 (10)	0.0269 (8)	
H214	0.3949	0.1352	0.0392	0.037 (4)*	
H215	0.5455	0.1314	0.0208	0.037 (4)*	
H216	0.4945	0.2419	0.0419	0.037 (4)*	
C214	0.4711 (3)	0.1641 (3)	0.11074 (10)	0.0259 (8)	
H217	0.4750	0.2460	0.1097	0.037 (4)*	
H218	0.5124	0.1381	0.1340	0.037 (4)*	
H219	0.3744	0.1396	0.1095	0.037 (4)*	
C221	0.9969 (3)	0.1591 (3)	0.08795 (9)	0.0179 (6)	
C222	1.0614 (3)	0.2197 (3)	0.05478 (10)	0.0244 (7)	
H221	1.1624	0.2168	0.0567	0.030 (3)*	
H222	1.0310	0.2980	0.0546	0.030 (3)*	
H223	1.0321	0.1832	0.0317	0.030 (3)*	
C223	1.0396 (3)	0.0371 (3)	0.08825 (9)	0.0242 (7)	
H224	1.0102	0.0014	0.0651	0.030 (3)*	
H225	0.9959	−0.0009	0.1093	0.030 (3)*	
H226	1.1404	0.0319	0.0906	0.030 (3)*	
C224	1.0400 (3)	0.2158 (3)	0.12421 (10)	0.0253 (8)	
H227	0.9974	0.1767	0.1452	0.030 (3)*	
H228	1.0092	0.2940	0.1240	0.030 (3)*	
H229	1.1409	0.2132	0.1266	0.030 (3)*	
N2	0.7361 (3)	0.3049 (2)	0.12375 (8)	0.0188 (6)	
C2	0.7134 (3)	0.3682 (3)	0.14610 (9)	0.0162 (6)	
S2	0.68141 (10)	0.46205 (7)	0.17794 (2)	0.0266 (2)	
N3	0.7594 (2)	0.3092 (2)	0.04267 (7)	0.0165 (5)	
C3	0.7802 (3)	0.3744 (3)	0.02045 (9)	0.0169 (6)	
S3	0.80855 (9)	0.46981 (7)	−0.01082 (2)	0.02275 (18)	

### Di-tert-butyldiisothiocyanatotin(IV) Atomic displacement parameters (Å^2^)

**Table:**

	*U*^11^	*U*^22^	*U*^33^	*U*^12^	*U*^13^	*U*^23^
Sn1	0.01571 (14)	0.01359 (16)	0.01288 (16)	0.00193 (11)	0.000	0.000
C111	0.0151 (19)	0.014 (2)	0.026 (3)	0.0000 (17)	0.000	0.000
C112	0.022 (2)	0.019 (2)	0.025 (3)	0.0045 (18)	0.000	0.000
C113	0.0204 (15)	0.0235 (18)	0.035 (2)	0.0015 (13)	−0.0095 (14)	−0.0094 (16)
C121	0.025 (2)	0.019 (2)	0.018 (2)	−0.0062 (18)	0.000	0.000
C122	0.0235 (15)	0.035 (2)	0.0227 (18)	−0.0030 (14)	0.0054 (14)	0.0016 (16)
C123	0.035 (3)	0.025 (3)	0.030 (3)	−0.010 (2)	0.000	0.000
N1	0.0214 (12)	0.0194 (15)	0.0152 (14)	0.0004 (11)	−0.0017 (10)	0.0047 (11)
C1	0.0181 (14)	0.0176 (16)	0.0162 (16)	0.0007 (12)	−0.0007 (12)	0.0057 (14)
S1	0.0499 (5)	0.0171 (4)	0.0147 (4)	0.0030 (4)	0.0006 (4)	−0.0018 (3)
Sn2	0.01568 (10)	0.01317 (11)	0.01369 (12)	0.00127 (8)	−0.00043 (7)	−0.00038 (8)
C211	0.0165 (14)	0.0216 (17)	0.0216 (17)	−0.0045 (12)	0.0005 (12)	0.0006 (14)
C212	0.0357 (19)	0.027 (2)	0.030 (2)	−0.0129 (16)	0.0020 (16)	−0.0025 (16)
C213	0.0198 (15)	0.039 (2)	0.0220 (18)	−0.0028 (14)	−0.0047 (13)	0.0009 (16)
C214	0.0218 (15)	0.032 (2)	0.0239 (19)	−0.0044 (14)	0.0062 (13)	0.0030 (15)
C221	0.0159 (13)	0.0193 (16)	0.0184 (17)	0.0027 (12)	−0.0013 (12)	−0.0033 (13)
C222	0.0194 (15)	0.0260 (19)	0.028 (2)	0.0009 (13)	0.0052 (14)	0.0009 (15)
C223	0.0262 (17)	0.0254 (19)	0.0211 (18)	0.0105 (14)	−0.0016 (13)	−0.0025 (14)
C224	0.0207 (16)	0.0287 (19)	0.0264 (19)	0.0039 (13)	−0.0053 (13)	−0.0094 (15)
N2	0.0205 (12)	0.0184 (14)	0.0176 (15)	−0.0020 (10)	−0.0025 (11)	0.0044 (12)
C2	0.0175 (14)	0.0151 (15)	0.0159 (17)	0.0008 (12)	−0.0010 (12)	0.0079 (13)
S2	0.0458 (5)	0.0190 (4)	0.0149 (4)	0.0104 (4)	0.0003 (4)	−0.0016 (3)
N3	0.0167 (12)	0.0187 (14)	0.0140 (14)	0.0002 (10)	−0.0017 (10)	−0.0032 (11)
C3	0.0159 (13)	0.0163 (16)	0.0185 (17)	0.0025 (12)	−0.0020 (12)	−0.0079 (13)
S3	0.0336 (4)	0.0198 (4)	0.0149 (4)	−0.0062 (3)	0.0004 (3)	0.0011 (3)

### Di-tert-butyldiisothiocyanatotin(IV) Geometric parameters (Å, º)

**Table:**

Sn1—N1^i^	2.180 (3)	Sn2—C221	2.227 (3)
Sn1—N1	2.180 (3)	C211—C212	1.525 (5)
Sn2—N2	2.172 (3)	C211—C213	1.526 (5)
Sn2—N3	2.175 (3)	C211—C214	1.526 (4)
Sn1—C121	2.214 (4)	C212—H211	0.9800
Sn1—C111	2.224 (4)	C212—H212	0.9800
S1—Sn2	3.1519 (9)	C212—H213	0.9800
S2—Sn1	3.1312 (9)	C213—H214	0.9800
S3—Sn2^ii^	3.1355 (9)	C213—H215	0.9800
C111—C113	1.524 (4)	C213—H216	0.9800
C111—C113^i^	1.524 (4)	C214—H217	0.9800
C111—C112	1.529 (6)	C214—H218	0.9800
C112—H111	0.9800	C214—H219	0.9800
C112—H112	0.9799	C221—C223	1.514 (4)
C113—H114	0.9800	C221—C224	1.529 (4)
C113—H115	0.9800	C221—C222	1.530 (4)
C113—H116	0.9800	C222—H221	0.9800
C121—C123	1.514 (7)	C222—H222	0.9800
C121—C122^i^	1.529 (4)	C222—H223	0.9800
C121—C122	1.530 (4)	C223—H224	0.9800
C122—H124	0.9800	C223—H225	0.9800
C122—H125	0.9800	C223—H226	0.9800
C122—H126	0.9800	C224—H227	0.9800
C123—H121	0.9800	C224—H228	0.9800
C123—H122	0.9800	C224—H229	0.9800
C123—H123	0.9800	N2—C2	1.126 (4)
N1—C1	1.130 (4)	C2—S2	1.633 (3)
C1—S1	1.625 (3)	N3—C3	1.134 (4)
Sn2—C211	2.217 (3)	C3—S3	1.625 (3)
			
N1^i^—Sn1—N1	84.35 (13)	C211—Sn2—S1	83.05 (9)
N1^i^—Sn1—C121	98.45 (11)	C221—Sn2—S1	84.07 (8)
N1—Sn1—C121	98.45 (11)	C212—C211—C213	110.3 (3)
N1^i^—Sn1—C111	98.31 (10)	C212—C211—C214	110.5 (3)
N1—Sn1—C111	98.31 (10)	C213—C211—C214	109.5 (3)
C121—Sn1—C111	157.3 (2)	C212—C211—Sn2	110.8 (2)
N1^i^—Sn1—S2	166.19 (7)	C213—C211—Sn2	108.4 (2)
N1—Sn1—S2	81.84 (7)	C214—C211—Sn2	107.2 (2)
C121—Sn1—S2	83.30 (7)	C211—C212—H211	109.5
C111—Sn1—S2	84.06 (6)	C211—C212—H212	109.5
C113—C111—C113^i^	110.8 (4)	H211—C212—H212	109.5
C113—C111—C112	110.0 (2)	C211—C212—H213	109.5
C113^i^—C111—C112	110.0 (2)	H211—C212—H213	109.5
C113—C111—Sn1	108.0 (2)	H212—C212—H213	109.5
C113^i^—C111—Sn1	108.0 (2)	C211—C213—H214	109.5
C112—C111—Sn1	110.1 (3)	C211—C213—H215	109.5
C111—C112—H111	109.2	H214—C213—H215	109.5
C111—C112—H112	109.2	C211—C213—H216	109.5
H111—C112—H112	109.4	H214—C213—H216	109.5
C111—C113—H114	109.5	H215—C213—H216	109.5
C111—C113—H115	109.5	C211—C214—H217	109.5
H114—C113—H115	109.5	C211—C214—H218	109.5
C111—C113—H116	109.5	H217—C214—H218	109.5
H114—C113—H116	109.5	C211—C214—H219	109.5
H115—C113—H116	109.5	H217—C214—H219	109.5
C123—C121—C122^i^	110.2 (3)	H218—C214—H219	109.5
C123—C121—C122	110.2 (3)	C223—C221—C224	110.2 (3)
C122^i^—C121—C122	109.2 (4)	C223—C221—C222	110.5 (3)
C123—C121—Sn1	112.1 (3)	C224—C221—C222	110.2 (3)
C122^i^—C121—Sn1	107.5 (2)	C223—C221—Sn2	110.3 (2)
C122—C121—Sn1	107.5 (2)	C224—C221—Sn2	107.8 (2)
C121—C122—H124	109.5	C222—C221—Sn2	107.8 (2)
C121—C122—H125	109.5	C221—C222—H221	109.5
H124—C122—H125	109.5	C221—C222—H222	109.5
C121—C122—H126	109.5	H221—C222—H222	109.5
H124—C122—H126	109.5	C221—C222—H223	109.5
H125—C122—H126	109.5	H221—C222—H223	109.5
C121—C123—H121	109.5	H222—C222—H223	109.5
C121—C123—H122	109.5	C221—C223—H224	109.5
H121—C123—H122	109.5	C221—C223—H225	109.5
C121—C123—H123	109.5	H224—C223—H225	109.5
H121—C123—H123	109.5	C221—C223—H226	109.5
H122—C123—H123	109.5	H224—C223—H226	109.5
C1—N1—Sn1	174.6 (2)	H225—C223—H226	109.5
C1—S1—Sn2	100.2 (1)	C221—C224—H227	109.5
C2—N2—Sn2	175.2 (3)	C221—C224—H228	109.5
C3—N3—Sn2	167.0 (2)	H227—C224—H228	109.5
N2—Sn2—N3	84.73 (10)	C221—C224—H229	109.5
N2—Sn2—C211	98.51 (11)	H227—C224—H229	109.5
N3—Sn2—C211	98.41 (10)	H228—C224—H229	109.5
N2—Sn2—C221	98.13 (10)	N1—C1—S1	178.2 (3)
N3—Sn2—C221	98.59 (10)	N2—C2—S2	178.8 (3)
C211—Sn2—C221	157.2 (1)	C2—S2—Sn1	100.7 (1)
N2—Sn2—S1	81.48 (7)	N3—C3—S3	178.8 (3)
N3—Sn2—S1	166.20 (7)	C3—S3—Sn2^ii^	99.8 (1)

Symmetry codes: (i) *x*, *y*, −*z*+1/2; (ii) *x*, −*y*+1/2, −*z*.

## References

[bb1] Alcock, N. W. & Sawyer, J. F. (1977). *J. Chem. Soc., Dalton Trans*, pp. 1090–1095.

[bb2] Allen, F. H., Kennard, O., Watson, D. G., Brammer, L., Orpen, A. G. & Taylor, R. (1987). *J. Chem. Soc. Perkin Trans. 2*, pp. S1–S19.

[bb3] Brandenburg, K. (2006). *DIAMOND*. Crystal Impact GbR, Bonn, Germany. Bruker (2019).

[bb4] Britton, D. (2006). *Acta Cryst.* C**62**, m93–m94. 10.1107/S0108270106002034 16518039

[bb5] Bruker (2009). *APEX2* and *SAINT*. Bruker AXS Inc., Madison, Wisconsin, USA.

[bb6] Cordero, B., Gómez, V., Platero-Prats, A. E., Revés, M., Echeverría, J., Cremades, E., Barragán, F. & Alvarez, S. (2008). *Dalton Trans.* pp. 2832–2838. 10.1039/b801115j 18478144

[bb7] Dakternieks, D., Jurkschat, K. & Tiekink, E. R. T. (1994). *Main Group Met. Chem.* **17**, 471–480.

[bb8] Kandil, S. A. & Allred, A. L. (1970). *J. Chem. Soc. A*, pp. 2987–2992.

[bb9] Krause, L., Herbst-Irmer, R., Sheldrick, G. M. & Stalke, D. (2015). *J. Appl. Cryst.* **48**, 3–10. 10.1107/S1600576714022985 PMC4453166 26089746

[bb10] Macrae, C. F., Sovago, I., Cottrell, S. J., Galek, P. T. A., McCabe, P., Pidcock, E., Platings, M., Shields, G. P., Stevens, J. S., Towler, M. & Wood, P. A. (2020). *J. Appl. Cryst.* **53**, 226–235. 10.1107/S1600576719014092 PMC6998782 32047413

[bb11] Mantina, M., Chamberlin, A. C., Valero, R., Cramer, C. J. & Truhlar, D. G. (2009). *J. Phys. Chem. A*, **113**, 5806–5812. 10.1021/jp8111556 PMC3658832 19382751

[bb12] Pancratz, A.-K., Kamrowski, A. & Reuter, H. (2024). *IUCrData*, **9**, x241093. 10.1107/S2414314624010939 PMC11618869 39649092

[bb13] Povray (2004). *POVRAY*. Persistence of Vision Pty. Ltd., Williamstown, Victoria, Australia.

[bb14] Reuter, H. & Pawlak, R. (2001). *Z. Kristallogr.* **216**, 56–59.

[bb15] Sawyer, J. F. (1988). *Acta Cryst.* C**44**, 633–636.

[bb16] Sheldrick, G. M. (2008). *Acta Cryst.* A**64**, 112–122. 10.1107/S0108767307043930 18156677

[bb17] Sheldrick, G. M. (2015). *Acta Cryst.* C**71**, 3–8.

[bb18] Westrip, S. P. (2010). *J. Appl. Cryst.* **43**, 920–925.

[bb19] Ye, F. & Reuter, H. (2012). *Acta Cryst.* C**68**, m104–m108. 10.1107/S0108270112012504 22476138

